# Standardization of the normative group for the third version of the test of visual attention – TAVIS

**DOI:** 10.1590/S1980-57642009DN20100005

**Published:** 2008

**Authors:** Gabriel Coutinho, Paulo Mattos, Catia Araújo, Manuela Borges, Angela Alfano

**Affiliations:** 1 Psicólogo do Centro de Neuropsicologia Aplicada, mestrando em saúde mental – Instituto de Psiquiatria da UFRJ.; 2 Psiquiatra, doutorado em Psiquiatria, Professor Associado – Instituto de Psiquiatria da UFRJ.; 3 Neuropsicóloga do Centro de Neuropsicologia Aplicada, Mestre em Informática – Instituto de Matemática da UFRJ.; 4 Psicóloga do Centro de Neuropsicologia Aplicada, Doutoranda em Psicologia – Instituto de Psicologia da UFRJ.

**Keywords:** attention, neuropsychology, computerized tests, atenção, neuropsicologia, testes computadorizados

## Abstract

Despite the importance of objective measures of attention to clinical practice, there is a paucity of Brazilian standardized tests.

**Objective:**

The aim of the present study was the standardization of a normative group for the third version of a computerized test of visual attention (TAVIS-3), developed to evaluate children and adolescents in the 6 to 17 years age range.

**Methods:**

631 students from 3 schools in Rio de Janeiro city were assessed with TAVIS-3, administered by experienced psychologists, following parents’ authorization.

**Results:**

The normative groups were determined considering performance of different age groups: from 6 to 10 years (with scores for 5 different ages); from 11 to 12 and from 13 to 17 years. Three tasks (focused, shifted and sustained attention) were standardized for each age group.

**Conclusion:**

The standardization of a normative group for TAVIS-3 discriminated performance ranges for distinct age groups, allowing its use as a neuropsychological assessment of attention.

## Abstract (pt)

Embora a avaliação objetiva da atenção seja de extrema importância para a prática clínica, observa-se escassez de testes neuropsicológicos com padronização brasileira para esta função.

**Objetivo:**

O objetivo do presente estudo foi desenvolver um grupo normativo de referência para a terceira versão de teste computadorizado de atenção visual (TAVIS-3), destinado à avaliação de crianças e adolescentes com idades entre 6 e 17 anos.

**Métodos:**

631 alunos de 3 instituições de ensino da cidade do Rio de Janeiro foram submetidos ao TAVIS-3, administrado por aplicadores treinados, mediante autorização dos pais.

**Resultados:**

A divisão dos grupos etários levando-se em consideração as diferenças de desempenho permitiu identificar faixas etárias de 6 aos 10 anos (com escores para cada uma das 5 idades compreendidas); de 11 a 12 anos e de 13 a 17 anos. Três tarefas distintas de atenção (seletiva, alternada e sustentada) foram padronizadas para cada faixa etária avaliada.

**Conclusões:**

A padronização de grupo normativo para o TAVIS-3 permitiu individualizar o desempenho de acordo com a idade, para uso como instrumento de avaliação neuropsicológica de atenção.

Attention has been conceived as a complex phenomenon that shares it limits with perceptual skills, memory, affect and consciousness.^1^ There are a number of different definitions of attention where the focused type has been the most used.^2^ The amount of information that may be processed simultaneously (working memory) is another commonly used definition for attention. It is noteworthy that attention is characterized by its variability even in the same individual.^3^

Attention is comprised of somewhat different aspects, as follows:

a) focused attention: is the ability to focus only on relevant stimulus despite distractive ones, selecting information relevant to conscious processing;
b) sustained attention: is the ability to sustain a consistent behavioral response through a continuous action. This skill comprises two different, but related, aspects: the amount of time that the performance level may be kept up, and performance consistency throughout this interval;
c) shifted attention: this is related to the ability of shifting focus among tasks with different cognitive ability demands, determining the information that will be focused on at any specific moment of time.

Attention deficit hyperactivity disorder (ADHD),^4^ Epilepsy^5^ and Learning Disorders (LD)^6^ are some of the most common neuropsychiatric disorders associated with inattention. The establishment of an accurate diagnosis of ADHD, for example, may demand an assessment with multiple informants (such as parents and teachers); however, some studies have demonstrated that the agreement rates between parents’ and teachers’ reports might be low.^7-9^ Therefore, objective measures of attention would prove extremely important to clinical practice.

Neuropsychological tests might provide two different, but related contributions:

a) contribute to the establishment of a clinical diagnosis;
b) determine the cognitive profile in cases where the clinical diagnosis had been previously determined, furnishing an estimate of the disorder’s severity and evolution, as well as response to treatments.^10^

The objective of the current paper was to work toward standardizing the normative data of a test previously developed in Brazil by Duchesne and Mattos^1^ to evaluate children and adolescents within the 6 to 17 years age, and now in its third version. This third version also differs from the former one because it has been adapted for Windows; other differences will be mentioned below. This computerized test consists of three tasks aimed at evaluating focused, shifted and sustained visual attention. Each of the tasks provides scores of hit reaction time, omission and commission errors for the aforementioned aspects. Both positive and negative predictive values of TAVIS-3 for Attention-Deficit Hyperactivity Disorder (ADHD) have been published elsewhere.^11^

## Methods

### Sample

631 consecutive children and adolescents with ages ranging from 6 to 17 years were evaluated over a 2-year period. Recruitment and assessment occurred in three schools (two private and one public) of Rio de Janeiro city. The evaluations were coordinated by schools’ educational services.

Individuals with previous history of epilepsy or traumatic brain injury and individuals undergoing treatment for psychiatric or neurological disorders were excluded from the study. Schools held data regarding students’ health and use of medication. Use of psychoactive substances was investigated with parents using direct questioning. The evaluations were conducted individually by intensively trained psychologists in quiet rooms at schools. The study was approved by the Ethics Committee of the Psychiatry Institute of the Federal University of Rio de Janeiro.

### Tools

TAVIS-3 included the following tasks:

#### Task 1

The examinee must selectively respond to target stimulus regardless of distracters. This task follows a model of tests that demands the subject to inhibit concurrent stimuli, often used in cancellation tests, such as the teste de *atenção concentrada* (AC)^12^. This task still requires visual scanning (ability to follow a group of information with eyes) as well as sustained attention. Impaired performances may suggest difficulties in focused or sustained attention.

#### Task 2

The examinee responds to the task shifting between two different patterns of rules: color/shape (12 year-old subjects or older) or equal/different (subjects younger than 12 years’ old). This task intends to evaluate shifted attention, demanding the subject to switch focus between two different concepts (adolescents) or two parameters of the same concept (children).

#### Task 3

This task demands the examinee to sustain attention for a certain amount of time (6 minutes for small children and 10 minutes for adolescents) while responding quickly to the appearance of a target stimulus. This is mainly a task of sustained attention, lasting for 10 minutes in adolescent form and 6 minutes for children.

In order to minimize difficulties due to little practice with the joystick, the examinee is previously trained with the tool. Also, each of the tasks comprises a training session in order to guarantee that a full understanding of the procedures was achieved. The training process of each task has a time limit that varies with age; however this process can be repeated in cases where examinee presents difficulties in understanding the instructions.

### Analyzed variables

The variables considered in the analysis of each of the tasks were:

#### Hit reaction time (HRT)

This is the time taken to respond to the stimulus. HRT is the average of the reaction times (throughout the task) and might be considered a measure of processing speed. Inattentive persons might appear to be attentive during the task; however the accuracy in detecting the stimulus is often reduced, with longer reaction time in comparison to normal subjects. This pattern has already been demonstrated in other etiologies^13-16^. When reaction time is very slow, there is the possibility of registering a “double error”, i.e., an omission error plus a subsequent commission error. This current version of TAVIS does not consider the subsequent commission error.

#### Omission errors (OE)

These occur when there is no register of a response to a target stimulus. This might occur due to the absence of a response to a target stimulus or if the response occurs only when the stimulus has already disappeared. Task 3 was developed to permit low frequency of omission errors.

#### Commission errors (CE)

This consists of responding to a distracter stimulus or in the absence of the target stimulus. In general, a high number of commissions may suggest impulsivity.

## Results

The sample was balanced for gender in all of the studied age groups. The number of children and adolescents submitted to each of the three tasks was not necessarily the same, given that task 2 was not administered to younger children and that some of the evaluations were not considered for the final analysis (Table 1).

**Table 1:** General characteristics of each task sample, divided by age group and gender.

	Total			Male			Female	
Age	N	%		N	%		N	%
**Task 1**								
6 7 8 9 10 11 to 12 13 to 17 Total	55 52 80 77 73 75 219 631	100.0 100.0 100.0 100.0 100.0 100.0 100.0 100.0		24 18 42 43 32 44 115 318	43.6 34.6 52.5 55.8 43.8 58.7 52.5 50.4		31 34 38 34 41 31 104 313	56.4 65.4 47.5 44.2 56.2 41.3 47.5 49.6
**Task 2**								
7 8 9 10 11 to 12 13 to 17 Total	42 41 47 51 58 105 344	100.0 100.0 100.0 100.0 100.0 100.0 100.0		14 20 27 20 28 56 165	33.3 48.8 57.4 39.2 48.3 53.3 48.0		28 21 20 31 30 49 179	66.7 51.2 42.6 60.8 51.7 46.7 52.0
**Task 3**								
6 7 8 9 10 11 to 12 13 to 17 Total	52 49 74 74 71 74 218 612	100.0 100.0 100.0 100.0 100.0 100.0 100.0 100.0		24 18 38 40 31 43 115 309	46.2 36.7 51.4 54.1 43.7 58.1 52.8 50.5		28 31 36 34 40 31 103 303	53.8 63.3 48.6 45.9 56.3 41.9 47.2 49.5

The normative age groups were determined considering the individual’s performance. Therefore, one group was created for those aged from 6 to 10 years; another group for ages 11 and 12; and another one for ages from 13 to 17 years. It is noteworthy that children aged from 6 to 7 years and 11 months were submitted to tasks in which stimuli were presented for a longer period. Only older children and adolescents were considered for task 2 due to the inconsistent and poor performance of younger children on this task. It is reasonable to consider that some of the difficulties faced by infants may be associated to reading abilities.

The descriptive statistics of HRT are shown in Table 2; the descriptive statistics of CE are shown in Table 3 and the descriptive statistics of OE are shown in Table 4.

**Table 2:** Hit reaction time (in seconds), divided by task and age group.

	Age						
	6	7	8	9	10	11 to 12	13 to 17
Task 1							
N Mean Standard deviation Minimum Maximum	55 0.743 0.090 0.510 0.930	52 0.673 0.077 0.470 0.890	80 0.555 0.051 0.434 0.700	77 0.540 0.055 0.388 0.670	73 0.500 0.050 0.375 0.611	75 0.470 0.052 0.340 0.580	219 0.449 0.036 0.361 0.553
**Task 2**							
N Mean Standard deviation Minimum Maximum		42 0.845 0.154 0.440 1.160	41 0.673 0.094 0.470 0.910	47 0.671 0.104 0.420 0.980	51 0.611 0.099 0.410 0.830	58 0.541 0.118 0.320 0.820	105 0.555 0.093 0.310 0.880
**Task 3**							
N Mean Standard deviation Minimum Maximum	52 0.641 0.134 0.410 0.920	49 0.585 0.082 0.440 0.800	74 0.492 0.090 0.340 0.760	74 0.464 0.086 0.335 0.735	71 0.421 0.057 0.309 0.590	74 0.388 0.064 0.276 0.570	218 0.357 0.048 0.253 0.510

**Table 3:** Number of commission errors (in percentage), divided by task and age group.

Commission errors	Age																			
	6			7			8			9			10			11-12			13-17	
	n	%		n	%		n	%		n	%		n	%		n	%		n	%
**Task 1**																				
0 1 2 3 4 5 6 7 8 9 10 to 16 Total	8 17 15 7 2 2 2 2 0 0 0 55	14.5 30.9 27.3 12.7 3.6 3.6 3.6 3.6 0.0 0.0 0.0 100		6 19 16 9 1 1 0 0 0 0 0 52	11.5 36.5 30.8 17.3 1.9 1.9 0.0 0.0 0.0 0.0 0.0 100		5 15 20 16 10 8 1 5 0 0 0 80	6.3 18.8 25.0 20.0 12.5 10.0 1.3 6.3 0.0 0.0 0.0 100		9 27 13 16 6 3 1 2 0 0 0 77	11.7 35.1 16.9 20.8 7.8 3.9 1.3 2.6 0.0 0.0 0.0 100		8 25 23 8 6 1 1 1 0 0 0 73	11.0 34.2 31.5 11.0 8.2 1.4 1.4 1.4 0.0 0.0 0.0 100		20 19 25 6 3 1 1 0 0 0 0 75	26.7 25.3 33.3 8.0 4.0 1.3 1.3 0.0 0.0 0.0 0.0 100		10 30 40 34 28 23 13 14 9 5 13 219	4.6 13.7 18.3 15.5 12.8 10.5 5.9 6.4 4.1 2.3 5.9 100
**Task 2**																				
0 1 2 3 4 5 6 7 8 9 10 to 13 Total				0 7 5 7 6 3 4 1 4 2 3 42	0.0 16.7 11.9 16.7 14.3 7.1 9.5 2.4 9.5 4.8 7.1 100		1 1 4 1 6 6 4 5 2 1 10 41	2.4 2.4 9.8 2.4 14.6 14.6 9.8 12.2 4.9 2.4 24.4 100		1 3 5 5 9 6 0 8 1 3 6 47	2.1 6.4 10.6 10.6 19.1 12.8 0.0 17.0 2.1 6.4 12.8 100		5 10 8 6 7 4 3 5 1 2 0 51	9.8 19.6 15.7 11.8 13.7 7.8 5.9 9.8 2.0 3.9 0.0 100		5 10 14 8 9 3 2 1 1 3 2 58	8.6 17.2 24.1 13.8 15.5 5.2 3.4 1.7 1.7 5.2 3.4 100		5 12 22 25 11 12 8 4 1 2 3 105	4.8 11.4 21.0 23.8 10.5 11.4 7.6 3.8 1.0 1.9 2.9 100
**Task 3**																				
0 1 2 3 4 5 6 Total	28 12 5 3 3 1 0 52	53.8 23.1 9.6 5.8 5.8 1.9 0.0 100		26 15 1 2 1 2 2 49	53.1 30.6 2.0 4.1 2.0 4.1 4.1 100		44 15 10 0 3 2 0 74	59.5 20.3 13.5 0.0 4.1 2.7 0.0 100		50 11 9 4 0 0 0 74	67.6 14.9 12.2 5.4 0.0 0.0 0.0 100		52 9 6 2 2 0 0 71	73.2 12.7 8.5 2.8 2.8 0.0 0.0 100		57 9 3 4 1 0 0 74	77.0 12.2 4.1 5.4 1.4 0.0 0.0 100		154 40 13 7 2 1 1 218	70.6 18.3 6.0 3.2 0.9 0.5 0.5 100

**Table 4:** Number of omission errors (in percentage). Divided by task and age group.

Omission errors	Age																			
	6			7			8			9			10			11-12			13-17	
	n	%		n	%		n	%		n	%		n	%		n	%		n	%
**Task 1**																				
0 1 2 3 4 5 6 7 8 9 to 16 Total	25 15 10 0 5 0 0 0 0 0 55	45.5 27.3 18.2 0.0 9.1 0.0 0.0 0.0 0.0 0.0 100		39 7 5 1 0 0 0 0 0 0 52	75.0 13.5 9.6 1.9 0.0 0.0 0.0 0.0 0.0 0.0 100		10 23 19 9 9 4 3 2 1 0 80	12.5 28.8 23.8 11.3 11.3 5.0 3.8 2.5 1.3 0.0 100		22 24 23 5 3 0 0 0 0 0 77	28.6 31.2 29.9 6.5 3.9 0.0 0.0 0.0 0.0 0.0 100		25 22 17 5 3 1 0 0 0 0 73	34.2 30.1 23.3 6.8 4.1 1.4 0.0 0.0 0.0 0.0 100		41 16 9 4 3 1 1 0 0 0 75	54.7 21.3 12.0 5.3 4.0 1.3 1.3 0.0 0.0 0.0 100		40 28 45 20 28 17 13 6 3 19 219	18.3 12.8 20.5 9.1 12.8 7.8 5.9 2.7 1.4 8.7 100
**Task 2**																				
0 1 2 3 4 5 6 Total				15 10 9 2 1 4 1 42	35.7 23.8 21.4 4.8 2.4 9.5 2.4 100		7 10 13 5 4 0 2 41	17.1 24.4 31.7 12.2 9.8 0.0 4.9 100		19 11 9 5 2 1 0 47	40.4 23.4 19.1 10.6 4.3 2.1 0.0 100		26 15 3 4 3 0 0 51	51.0 29.4 5.9 7.8 5.9 0.0 0.0 100		33 14 6 4 1 0 0 58	56.9 24.1 10.3 6.9 1.7 0.0 0.0 100		40 32 24 7 2 0 0 105	38.1 30.5 22.9 6.7 1.9 0.0 0.0 100
**Task 3**																				
0 1 Total	52 0 52	100 0.0 100		49 0 49	100 0.0 100		74 0 74	100 0.0 100		74 0 74	100 0.0 100		71 0 71	100 0.0 100		73 1 74	98.6 1.4 100		215 3 218	98.6 1.4 100

## Discussion

Given that attention is a complex phenomenon, it must be emphasized that TAVIS-3 allows the evaluation of different aspects of visual attention (focused, shifted and sustained) separately. Moreover, the test provides indexes of HIT, CE and OE for each of the three aforementioned aspects of visual attention; it also permits identification of deficits with high accuracy. The training session with the joystick before the administration of each of the tasks is especially important to guarantee the reliability of the results because it minimizes the possibility of poor performances due to low familiarity with the tools.

Mattos^17^ presented the most important advantages of utilizing computerized tests:

a) optimization of time (there is no need for consulting tables or calculating scores),
b) higher accuracy in administrating and correcting (both automatic),
c) the possibility to show complex stimulus on screen,
d) the possibility of obtaining specific variables (for example, hit reaction time in seconds, information that is often imprecise when measured manually with a chronometer),
e) frees the examiner to observe the examinee’s behavior qualitatively during the execution of the task.

Working with percentiles allows a dimensional analysis of the results, instead of classifying the performances only as normal or impaired. Thus, it is possible to estimate the *severity* of the impairment based on comparisons to normative groups.

Some limitations must be considered before administering and interpreting these results, as in any neuropsychological test, namely: important motor deficits, acromatopsy, daltonism, deficits in visual perceptual skills and behavioral variables through the execution of the tasks which might generate results with low reliability.

It must be noted that neuropsychological test results must be understood in the light of clinical history and also taking into consideration the neuropsychologist’s *qualitative* evaluation. Impaired performances in tests of visual attention are neither sufficient nor necessary for the diagnosis of any neuropsychiatric disorder. Nevertheless, when combined with clinical history and qualitative evaluation, the results of the test may be extremely useful to delineate the symptomatic profile, enabling a more precise diagnosis.

The standardization of the normative group for TAVIS-3 will allow its use as another tool for neuropsychological assessment. Although there are other tests of attention with Brazilian normalization – such as the AC^11^ and, more recently, the Stroop^18^ – there are currently few computerized tests available in our environment. The use of computerized tests decreases the chances of errors in administering and correcting them, which is very important to assure the reliability of the results obtained.

### Limitations

The current study should be understood in light of some limitations. We did not investigate the presence of LD in our sample, which might be considered a limitation given that previous studies have shown that LD may present with impairments in attention tasks 6. Also, adolescents were not questioned about psychoactive substances use; this issue was investigated only using parents’ reports. We did not compare the influence of school type (private or public) on test performance; however, the public school where the study was conducted is one of the best regarded in the city of Rio de Janeiro, which might have minimized the differences in task performances between students from public and private schools.

## Figures and Tables

**Figure 1:**
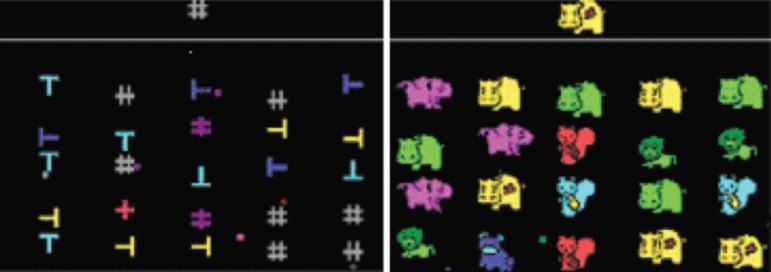
Task 1 Stimuli (geometric figures for adolescents, and animals for children). The examinee must press the button as soon as the target stimuli appear. Despite the distracters.

**Figure 2:**
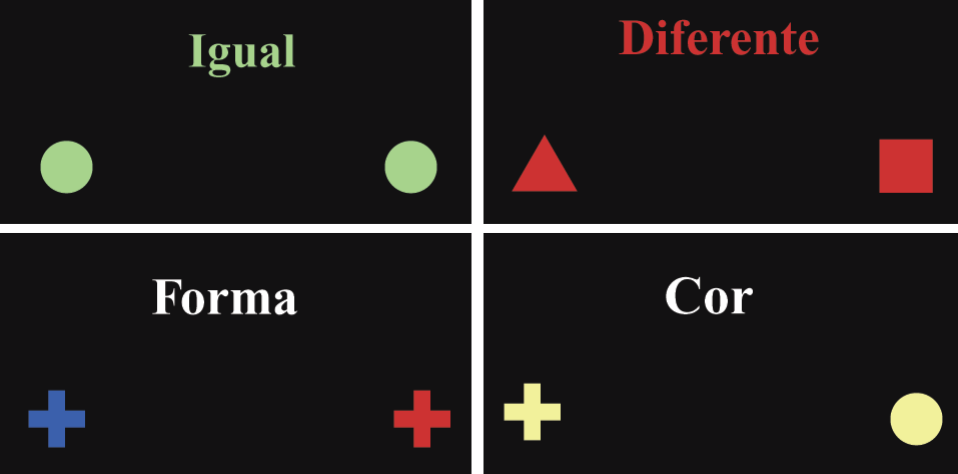
Concepts of Task 2 (Equal and different for children; and shape and color for adolescents). The examinee must press the button whenever the stimuli satisfy the rule.

**Figure 3:**
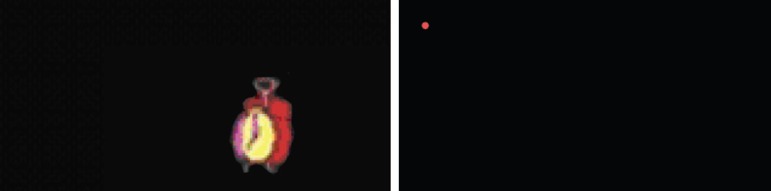
Task 3 stimuli for children. The examinee must press the button whenever a target stimulus appears in the empty screen, maintaining focus throughout a monotonous task.

## References

[1] Duchesne M, Mattos P (1997). Normatização de um teste computadorizado de
atenção visual. Arq Neuropsiquiatr.

[2] Nahas T, Xavier G, Andrade VM, Santos FH, Bueno OFA (2004). Atenção. Neuropsicologia Hoje.

[3] Lezak MD (2004). Neuropsychological assessment.

[4] American Academy of Pediatrics (2000). Clinical Practice Guideline: Diagnosis and Evaluation of the
Child with Attention-Deficit/Hyperactivity Disorder. Pediatrics.

[5] Schubert R (2005). Attention deficit disorder and epilepsy. Pediatr Neurol.

[6] Homack S, Riccio CA (2004). A meta-analysis of the sensitivity and specificity of the Stroop
Color and Word Test with children. Arch Clin Neuropsychol.

[7] Wolraich ML, Lambert EW, Bickman L, Simmons T, Doffing MA, Worley KA (2004). Assessing the Impact of Parent and Teacher Agreement on
Diagnosing Attention-Deficit Hyperactivity Disorder. Dev Behav Pediatrics.

[8] Sayal K, Taylor E (2005). Parent ratings of school behaviour in children at risk of
attention deficit/hyperactivity disorder. Acta Psychiatr Scand.

[9] Biederman J, Faraone SV, Monuteaux MC, Grossbard JR (2004). How Informative Are Parent Reports of
Attention-Deficit/Hyperactivity Disorder Symptoms for Assessing Outcome in
Clinical Trials of Long-Acting Treatments? A Pooled Analysis of Parents' and
Teachers' Reports. Pediatrics.

[10] Mattos P, Alfano A, Araujo C, Kapczinski F, Quevedo J, Izquierdo I (2004). Avaliação Neuropsicológica. Bases Biológicas dos Transtornos Psiquiátricos.

[11] Coutinho G, Mattos P, Araujo C, Duchesne M (2007). Transtorno do Déficit de Atenção e
Hiperatividade: Contribuição Diagnóstica de
Avaliação Computadorizada de Atenção
Visual. Rev Psiq Clín.

[12] Cambraia SV (2004). Teste de Atenção Concentrada.

[13] Gronwall D, Wrightson P (1974). Delayed recovery after minor head injury. Lancet.

[14] Van Zomeren AH, Deelman BG (1978). Long-term recovery of visual reaction time after closed head
injury. J Neurol Neurosurg Psychiatry.

[15] Collings RD (2003). Differences Between ADHD Inattentive and Combined Types on the
CPT. J Psychopathol Behav Assess.

[16] Nigg JT, Blaskey LG, Huang-Pollock CL, Rappley MD (2002). Neuropsychological Executive Functions and DSM-IV ADHD
Subtypes. J Am Acad Child Adolesc Psychiatry.

[17] Mattos P, Gagliardi RJ, Reimao R (1998). Uso de testes computadorizados em Neuropsicologia. Clínica Neurológica.

[18] Duncan MT (2006). Obtenção de dados normativos para desempenho no
teste de Stroop num grupo de estudantes do ensino fundamental em
Niterói. J Bras Psiq.

